# Comprehensive Assessment of Fish Diversity Based on Surface Water and Sediment eDNA—A Case from Sansha Bay, Southeastern China

**DOI:** 10.3390/biology15161336

**Published:** 2026-08-07

**Authors:** Zhi Zhang, Yuting Zhang, Zhongyuan Shen, Feipeng Wang, Jingli Mu

**Affiliations:** 1 Fujian Key Laboratory on Conservation and Sustainable Utilization of Marine Biodiversity, College of Geography and Oceanography, Minjiang University, Fuzhou 350108, China; ytzhang@mju.edu.cn (Y.Z.); fpwang@mju.edu.cn (F.W.); 2 Engineering Research Center of Polyploid Fish Reproduction and Breeding of the State Education Ministry, College of Life Sciences, Hunan Normal University, Changsha 410081, China; zyshen@hunnu.edu.cn

**Keywords:** fish diversity, environmental DNA, surface water, sediment, aquaculture bay

## Abstract

Shansha Bay, southeastern China, is a typical semi-enclosed bay facing intense anthropogenic pressure, with mariculture production reaching approximately 660,000 tons annually, predominantly driven by the large yellow croaker (*Larimichthys crocea*) industry. To assess how these pressures influence biodiversity detection, we employed eDNA metabarcoding targeting the 12S rRNA gene to survey fish diversity across 10 sites, integrating both surface water and sediment matrices. A total of 40 species belonging to 16 orders, 27 families and 32 genera, were identified. Among them, *L. crocea*, *Bostrychus sinensis*, *Clupanodon thrissa* and *Siganus fuscescens* are the dominate species, which comprised over half of all OTUs. Comparison with historical trawl surveys revealed 30 newly recorded species, including the Japanese eel (*Anguilla japonica*) and oarfish (*Regalecus glesne*). Alpha diversity was low (Shannon: 0.69–1.93), with significant spatial disparities driven by freshwater runoff and hydrodynamics. While eDNA effectively captured the dominance of cultured species like *L. crocea*, the extreme biomass signal poses challenges for detecting wild populations. Our findings underscore that a multi-matrix eDNA approach is crucial for accurate biodiversity assessments in aquaculture-dominated coastal ecosystems, although distinguishing wild stocks from aquaculture-derived DNA remains a critical methodological hurdle.

## Simple Summary

Monitoring fish biodiversity in coastal waters is challenging, especially in areas with intensive fish farming like Sansha Bay, China. This study used environmental DNA (eDNA) technology to survey fish by analyzing both water and sediment samples. We found that combining these two methods provided a more complete picture of marine life than using water samples alone. In total, we identified 40 fish species, including 30 species never before recorded in recent surveys of the bay. Notably, all five species of flatfishes (Pleuronectiformes), such as *Cynoglossus gracilis*, were detected exclusively in the sediment, proving that “mud sampling” is crucial for finding bottom-dwelling fish. While we detected DNA from rare species like the oarfish (*Regalecus glesne*) and the Japanese eel (*Anguilla japonica*), we caution that this might reflect DNA transported by currents or from aquaculture activities, rather than resident populations. Conversely, the high abundance of large yellow croaker (*Larimichthys crocea*) DNA largely reflected the bay’s massive aquaculture output. Our findings suggest that future monitoring programs should collect both water and sediment samples to accurately track fish diversity, especially in complex environments where traditional fishing nets often miss elusive species.

## 1. Introduction

Fish constitute a crucial component of aquatic ecosystems, providing not only abundant protein for humans but also maintaining ecosystem stability [1]. Over the past decade, research efforts have intensified to understand the dynamics of fish diversity, driven by increasing anthropogenic pressures and the need for effective conservation strategies [2,3]. Traditional methods for assessing fish diversity often suffer from limitations such as low detection rates of rare or elusive species, time and labor intensiveness, and destructive sampling practices [4]. Consequently, there is a pressing demand for innovative approaches that can overcome these challenges and provide accurate assessments of fish community structures [5].

Environmental DNA (eDNA) technology has emerged as a promising tool for biodiversity monitoring and assessment, revolutionizing the field of aquatic ecology [6,7]. By detecting genetic material shed by organisms into their surrounding environment, eDNA analysis offers several advantages, including high sensitivity, non-invasive sampling, and the ability to detect species that are difficult to capture using traditional methods [8]. eDNA has been widely applied in various aquatic environments, ranging from freshwater streams and lakes to marine ecosystems, facilitating comprehensive assessments of biodiversity and community composition [2,9].

Sansha Bay, located in southeastern China (26°30′–26°58′ N, 119°26′–120°10′ E), represents a unique and ecologically significant habitat for fish species, encompassing diverse habitats ranging from shallow nearshore waters to deeper offshore regions [10]. Covering an area of approximately 714 km^2^, the semi-enclosed bay serves as a dynamic interface between terrestrial and marine ecosystems. However, the bay’s ecological significance is inextricably linked to its role as one of China’s most intensive mariculture areas. Aquaculture activities currently utilize approximately 69% of the bay’s surface area, with the total mariculture output reaching 664,700 tons [11]. Over the past decade, the mariculture area has expanded from 24,354 ha to 30,348 ha, and production has increased from 329,400 tons to 566,100 tons [12], reflecting the rapid intensification of anthropogenic pressure in this coastal system. Sansha Bay is widely recognized as the “hometown” of large yellow croaker (*Larimichthys crocea*) [13,14,15,16]. The bar harbors the world’s largest concentration of *L. crocea* cage aquaculture, with approximately 210,000 cages currently in operation and cage-farming areas expanding from 9.1 km^2^ to 33.4 km^2^ over the past 15 years [12]. In addition to the croker, Sansha Bay supports the culture of other commercially important species, including the sea bass (*Lateolabrax japonicus*), red drum (*Sciaenops ocellatus*), and groupers (*Epinephelus* spp.). This intensive production is sustained by massive external inputs that millions of tons of feed are applied annually, with trash fish feed comprising approximately 80% of the total [17].

Previous studies have provided valuable insights into the fish diversity of Sansha Bay, identifying key species and highlighting the area’s importance as a fish spawning ground [18,19]. Previous studies, including a six-month survey in 2019, identified 58 fish species at 25 stations through morphological and DNA barcode identification methods [20]. In addition, few studies focused specifically on the fish diversity from Sansha Bay. Instead, more studies have tended to focus on the impact of aquaculture activities on the marine environment [10,21,22,23]. In the present study, we aim to address these knowledge gaps by eDNA to assess fish diversity and community structure in Sansha Bay, providing valuable insights for conservation and management initiatives. Through a comparative analysis of water and sediment samples, as well as comparison with traditional trawl catch data, we would like to find optimize sampling strategies for more accurate assessments of fish diversity in the future monitoring.

## 2. Materials and Methods

### 2.1. Sample Collection

**Figure 1:**
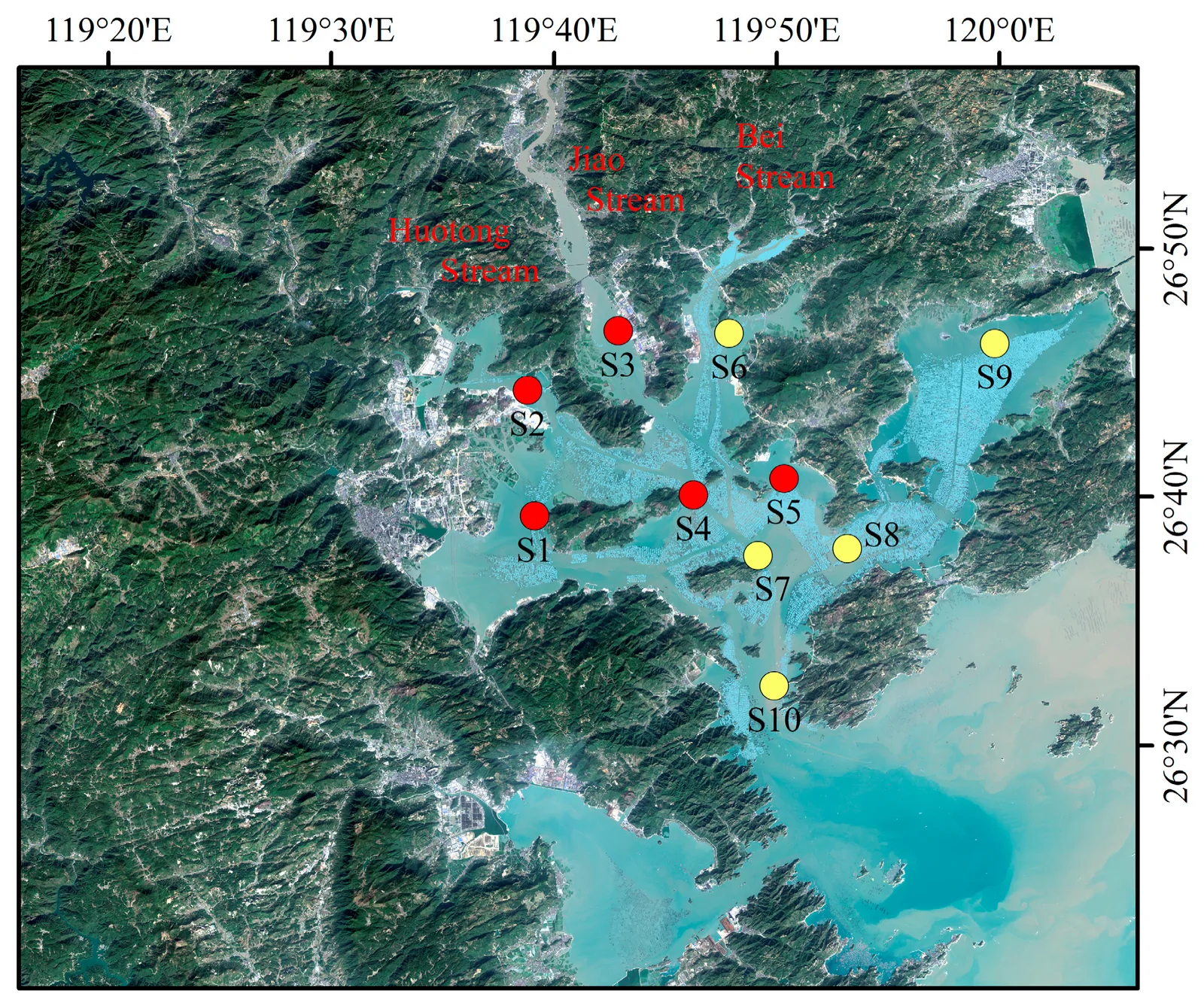
Sampling stations in this study. Five sites marked red (S1~S5) indicate that both water and sediment samples were collected, while the yellow sites (S6~S10) only collected water samples.

Sampling was conducted in April 2024 at 10 sites from Sansha Bay (Figure 1). The depth of the bay varies from 4 to 30 m, and the water temperature and salinity ranged from 14.07 to 16.42 and 14.78 to 28.93, respectively. As a semi-enclosed bay, there is only a mouth about 2.5 km wide connected to the outside. Sites were strategically selected based on a decade-long baseline dataset of hydrodynamics, substrate distribution, and fisheries productivity in the bay. We collected samples in S10 to evaluate the exchange of Sansha Bay with the open sea. The sites (S1, S2, S3 and S6) from estuary receive a large amount of freshwater rushing in from the dense channels during the rainy season. The navigation channel bears the pressure of busy traffic, including S4, S5 and S7. Finally, the Dongwuyang Bay, which has the most stable hydrology, is the most densely aquaculture zone, where nearly 10 percentage of China’s macroalgae production was cultivated. Surface water samples were collected from all sites, while sediment samples were obtained from five selected sites. Sediment sampling was restricted to these sites due to physical constraints that the outer bay is characterized by gravelly, consolidated substrates and deep outer-channel zones exceed the operational safety limits.

At each sampling site, three replicate samples were collected using a water sampler, with each sample containing 2 L of surface water and stored in polyethylene bottles. A benthic collector was used for sediment sampling, and more than 100 g of surface sediment was wrapped in tinfoil, brought back to the laboratory for freezing preservation, and DNA extraction was completed within 24 h. Sampling equipment and bottles were disinfected with a 10% (*w*/*v*) sodium hypochlorite solution. All water samples were filtered within 24 h using 0.45 μm mixed cellulose ester filters (Whatman, Little Chalfont, UK) under vacuum. Negative controls were included with each filtration to assess exogenous DNA contamination. After filtration, filters were placed in 4.5 mL nuclease-free cryotubes and frozen in liquid nitrogen. Filtration equipment was disinfected and rinsed to prevent cross-contamination between samples.

### 2.2. eDNA Extraction, PCR Amplification and Sequencing

For water samples, DNA extraction from the filters was performed using PowerWater DNA Isolation Kits (Qiagen, Hilden, Genmany) following the manufacturer’s instructions. For sediment samples, DNA was extracted by 500 mg sediment using FastDNA Spin Kit for Soil (MP Biomedicals, Irvine, CA, USA) following the manufacturer’s instructions. The quality of extracted DNA was assessed by 1% (*w*/*v*) agarose gel electrophoresis. Each sample was extracted independently, and a blank filter was included as a negative control. Extracted DNA samples were rapidly frozen at −20 °C until PCR amplification.

This study employed the MiFish-F (5′-ACTGGGATTAGATACCCC-3′) and MiFish-R (5′-TAGAACAGGCTCCTCTAG-3′) primers for fish eDNA metabarcoding analysis targeting the mitochondrial 12 s v5 gene [24]. The PCR reaction mixture (20 μL) contained 2–5 μL template DNA (10 ng/μL), 0.8 μL each of forward and reverse primers (5 μmol/L), 2 μL dNTPs, 4 μL 5× FastPfu buffer, 0.4 μL FastPfu polymerase, and was supplemented with ddH2O to a final volume of 20 μL. The PCR amplification protocol included an initial denaturation at 95 °C for 5 min, followed by 35 cycles of denaturation at 95 °C for 30 s, annealing at 55 °C for 30 s, extension at 72 °C for 45 s, and a final extension at 72 °C for 10 min. Three replicate samples from each site were extracted and amplified, separately. Then the resulting amplicons from the same site were pooled. Gel electrophoresis with 2% (*w*/*v*) agarose was used to verify the PCR products. We observed distinct primer-dimer bands (<100 bp) in preliminary gels. While magnetic bead cleanup is standard, we opted for gel extraction specifically to exclude these short fragments and ensure only the target ~170 bp 12 s fragment was sequenced. A single, clear band of expected size was observed for the vast majority of samples. Library preparation and sequencing were outsourced to Shanghai LingEn Biotechnology Co., Ltd. (Shanghai, China) Libraries were quantified using Qubit (dsDNA HS Assay, Waltham, MA, USA) and qPCR, then normalized prior to sequencing. Paired-end sequencing (2 × 250 bp) was performed on an Illumina NovaSeq 6000 platform.

### 2.3. Data Analysis

Sequencing reads were quality-filtered and assembled to obtain high-quality sequences for each sample. Operational taxonomic units (OTUs) were clustered at a similarity threshold of ≥97%, and representative sequences were compared and annotated against the Mito database (v4.03, https://mitofish.aori.u-tokyo.ac.jp/mifish/ accessed on 25 June 2026) for taxonomic identification [25,26]. Based on the OTU clustering and annotation results, species composition and alpha diversity analysis were performed. OTU sequences were matched against the database to determine fish species information, with a threshold condition of Identity value ≥ 97% and E-value < 10^−5^. Fish taxonomic information was referenced from the Fishbase database (www.fishbase.org accessed on 25 June 2026) and Fujian Fish Fauna [27].

Alpha diversity analysis included Chao1 index, Shannon index, Simpson index, and Coverage index to assess community richness, diversity, and coverage. Principal coordinates analysis (PCoA) based on Bray–Curtis distance matrix was conducted using the R software vegan package v2.6-6.1 to explore spatial distribution differences in community composition among different sampling sites [28]. Additionally, species diversity results from sediment and water samples were compared, and eDNA data were compared with traditional trawl catch data to demonstrate the accuracy of eDNA methods and propose optimization suggestions.

## 3. Results

### 3.1. Subsection

**Figure 2:**
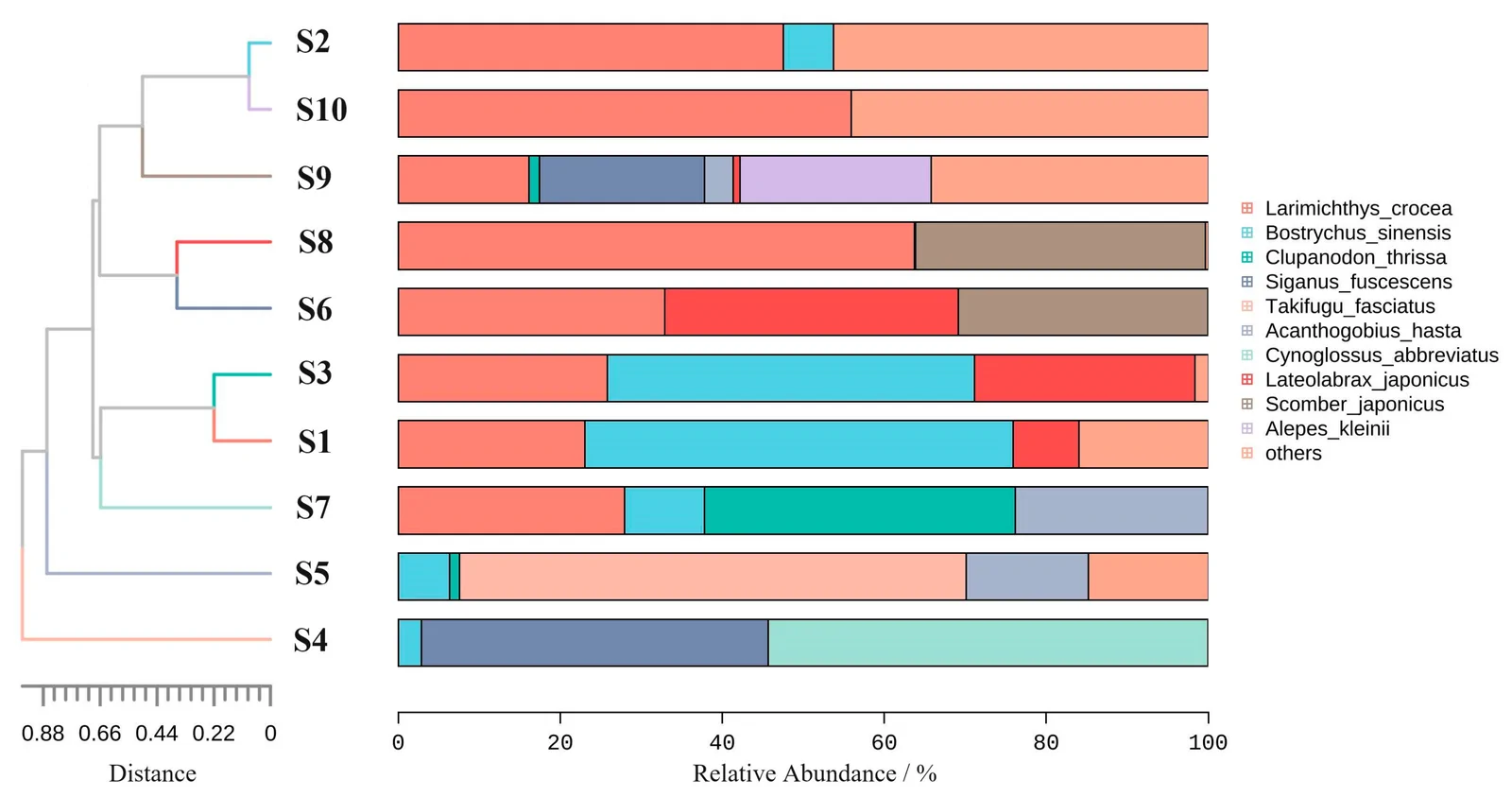
Composition and relative abundance of fish species at each sampling site (with their phylogeny by maximum intraspecific distance).

A total of 2,228,404 reads set to 229 ± 25 bp length, were obtained from 10 sampling sites, with the highest number of OTUs recorded at site S7 (49,180) and the lowest at S10 (4509). After annotation, a total of 40 fish species belonging to 16 orders, 27 families, and 32 genera were identified. The distribution of species and sequence numbers at each sampling site is presented in Table 1. Order Perciformes accounted for the highest proportion of species (9 out of 40), followed by the order Pleuronectiformes (6 out of 40). Based on sequence abundance at each sampling site, the species composition and relative sequence abundance are depicted in Figure 2. The dominate species detected by eDNA metabarcoding were *Larimichthys crocea*, *Bostrychus sinensis*, *Clupanodon thrissa* and *Siganus fuscescens*.

**Table 1:** Species composition and OTU distribution of each sampling site.

Species	S1	S2	S3	S4	S5	S6	S7	S8	S9	S10
Anguillidae										
*Anguilla japonica*	0	0	2	0	0	0	0	0	0	0
Engraulidae										
*Coilia mystus*	0	0	0	2	1	0	0	0	4	0
*Coilia grayii*	0	0	0	0	6	0	0	0	6	0
*Engraulis* sp.	0	0	0	0	4207	0	0	2	0	0
Dorosomatidae										
*Clupanodon thrissa*	0	0	0	0	358	0	18,869	0	402	0
Myctophidae										
*Benthosema pterotum*	0	0	0	0	0	0	0	0	0	1986
Lateolabracidae										
*Lateolabrax japonicus*	1140	0	3819	0	0	9277	0	0	263	0
Carangidae										
*Alepes djedaba*	0	0	0	0	0	0	0	0	5	0
*Alepes kleinii*	0	0	0	0	0	0	0	6	7401	0
*Megalaspis cordyla*	0	5	0	0	0	0	0	0	0	0
Leiognathidae										
*Photopectoralis bindus*	1438	4551	0	0	1	0	0	4	0	0
Epinephelidae										
*Plectropomus leopardus*	0	0	0	0	2	0	0	17	0	0
Scatophagidae										
*Scatophagus argus*	0	0	0	0	0	0	0	7	0	0
Sparidae										
*Evynnis tumifrons*	27	0	96	0	0	0	0	0	0	0
Acropomatidae										
*Acropoma japonicum*	0	0	0	0	0	0	0	12	0	0
Sciaenidae										
*Collichthys lucidus*	4	4	2	7	7	3	10	4	5045	0
*Larimichthys crocea*	3240	6929	3629	1	0	8407	13,745	11,484	5061	2523
*Atrobucca nibe*	0	0	0	0	0	0	0	0	3	0
*Larimichthys polyactis*	0	4	0	0	0	0	4	0	6	0
Blenniidae										
*Omobranchus elegans*	1	1	0	0	0	0	0	0	0	0
Terapontidae										
*Terapon theraps*	0	0	0	0	0	0	0	0	1753	0
Rachycentridae										
*Rachycentron canadum*	0	0	0	0	0	0	0	5	0	0
Butidae										
*Bostrychus sinensis*	7444	900	6367	802	1844	0	4843	2	0	0
Eletridae										
*Eleotris oxycephala*	665	2163	0	0	0	0	0	0	0	0
Oxudercidae										
*Acanthogobius hasta*	0	0	0	0	4359	0	11,707	0	1103	0
Gobiidae										
*Yongeichthys viganensis*	0	0	0	0	0	0	0	8	0	0
*Istigobius campbelli*	0	0	0	0	0	0	0	0	4	0
*Parachaeturichthys polynema*	0	0	0	0	0	0	0	2	3621	0
Siganidae										
*Siganus fuscescens*	0	0	0	12,032	0	0	2	21	6402	0
*Siganus spinus*	0	0	0	0	0	0	0	0	2	0
*Siganus canaliculatus*	0	0	0	0	0	0	0	0	275	0
Regalecidae										
*Regalecus glesne*	6	7	2	0	1	1	0	1	3	0
Scombridae										
*Scomber japonicus*	0	0	0	0	0	7883	0	6436	3	0
Cynoglossidae										
*Cynoglossus gracilis*	0	0	0	2	0	0	0	0	0	0
*Cynoglossus abbreviatus*	0	0	0	15,259	0	0	0	0	0	0
*Paraplagusia japonica*	104	0	129	7	0	0	0	0	0	0
Tetraodontidae										
*Lagocephalus cheesemanii*	0	0	0	0	33	0	0	0	0	0
*Takifugu alboplumbeus*	0	0	0	0	5	0	0	0	0	0
*Takifugu bimaculatus*	0	0	0	0	4	0	0	0	0	0
*Takifugu xanthopterus*	0	0	0	0	27	0	0	0	0	0

Note: Values represent OUT counts retained after quality filtering and annotation against the MitoFish database. For species composition analysis, S1 to S5 included both water and sediment results. But sediment data were not used to subsequently analyze diversity indices (Table 2).

**Table 2:** Alpha diversity index of each site.

Sampling Site	Richness	Shannon	Simpson	Pielou	Chao1	Good’s Coverage
S1	10	1.31	0.64	0.57	10	0.9929
S2	9	1.18	0.65	0.54	9	0.9931
S3	8	1.14	0.65	0.55	8	1.0000
S4	8	0.80	0.52	0.39	8	0.9964
S5	15	1.11	0.56	0.41	16.5	0.9896
S6	5	1.10	0.67	0.68	5	0.9961
S7	7	1.30	0.71	0.67	7	1.0000
S8	15	0.69	0.47	0.26	15	0.9944
S9	19	1.93	0.83	0.66	19	1.0000
S10	2	0.69	0.49	0.99	2	1.0000

Comparing our findings with historical data, fish surveys in Sansha Bay were reported mainly in the 1990s, 2008, and 2019. Previous surveys focused on traditional trawl catches, with eDNA analysis being conducted for the first time in this study. A comparison revealed significant differences, with 30 newly recorded species identified through eDNA analysis. However, substantial disparities were found between each survey (Supplementary Table S1) [18,20,29,30]. Among these four surveys, 11, 20, 39 and 30 species endemic to only one survey period were detected, which accounts for three-quarters of the total species that have ever appeared in this region.

### 3.2. Diversity Index

Alpha diversity analysis of fish communities at different stations in Sansha Bay was conducted, and the Chao1, Shannon, and Simpson diversity indices, along with Good’s coverage values, are presented in Table 2. The station S9 exhibited the highest Shannon–Wiener index (1.93), followed by S1 (1.31), while S10 showed the lowest value (0.69). The Simpson index was highest at S9 (0.83), followed by S7 (0.70), and lowest at S8 (0.47). The coverage value reported herein refer to Good’s coverage, which estimates the completeness of the sequencing effort in capturing the species present in the sample. Rather than representing physical genomic coverage, it calculates the probability that a randomly selected read from the sample belongs to a species already represented in the dataset. Here, Good’s coverage values for all samples ranged from 0.9896 to 1.0000, indicating that the sequencing depth was sufficient to capture the vast majority of the fish diversity present and that the proportion of undetected taxa was negligible.

**Figure 3:**
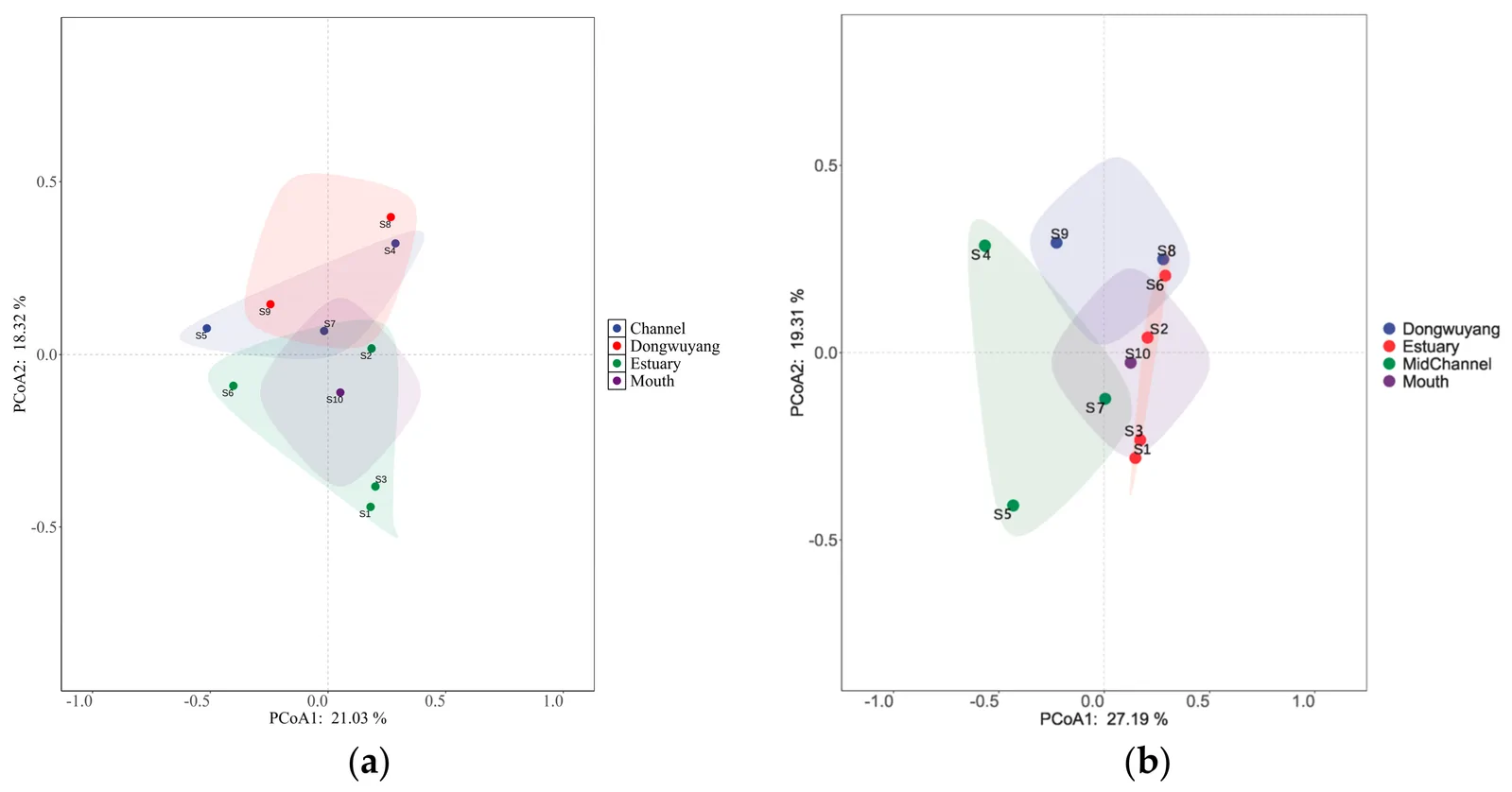
Principal coordinates based on (**a**) Jaccard distance and (**b**) Bray–Curtis distance matrix analysis of fish detected by environmental DNA.

Based on the sequence abundance at each sampling site, PCoA was performed using both Jaccard (presence/absence) and Bray–Curtis (abundance) distance matrices to evaluate beta diversity (Figure 3). The Jaccard-based PCoA revealed considerable overlap between samples from the navigation channel and the inner Dongwuyang Bay, indicating high similarity in species composition. Notably, S7 occupied a central position in the ordination space, reflecting its geographical location at the intersection of different hydrological regimes. This suggests that frequent water exchange facilitates species connectivity between these regions. In contrast, the Bray–Curtis analysis, which incorporates read abundance, showed a slightly different pattern. In contrast, the Bray–Curtis distance, which incorporates species abundance, highlighted a clear gradient along PCoA1 separating the high-abundance Dongwuyang and Channel stations from the lower Estuary stations. Moreover, S10 from mouth of Sansha Bay diverged significantly from other Channel stations, suggesting that despite similar species identities, the relative abundance and biomass of fish communities varied considerably between the confined mid-channel waters and the dynamic tidal mouth area. Overall, the distinct clustering patterns observed under both metrics confirm significant spatial heterogeneity in the fish community structure of Sansha Bay.

### 3.3. Comparison Between Water and Sediment Samples

**Figure 4:**
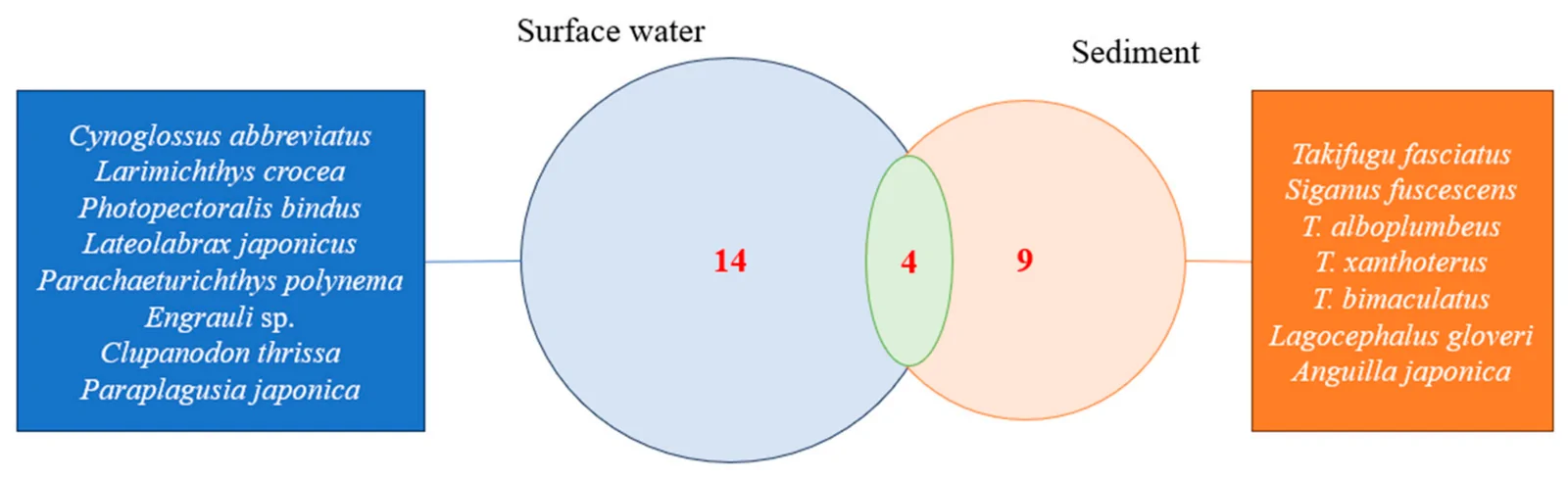
Comparison of eDNA identification between water and sediment samples.

Surface water and sediment samples were simultaneously collected at five sites (S1, S2, S3, S4, and S5), resulting in the detection of 27 fish species. Among these, 14 species were unique to surface water, nine were unique to sediment, and only four species were common to both (Figure 4). Furthermore, in terms of species composition, several species such as *C. thrissa* and *L. crocea* were abundant in surface water samples (OTUs > 4000), while sediment-specific species included *C. thrissa* and *B. sinensis*. Additionally, all five species of Pleuronectiformes were exclusively detected in sediment eDNA. Notably, sediment eDNA analysis also revealed the presence of protected species such as *Anguilla japonica*.

## 4. Discussion

In the present study, employed eDNA technology, a comprehensive analysis of fish diversity and community structure in Sansha Bay, southeastern China was conducted. The results unveiled a rich diversity of fish species, with 30 previously unreported species detected. Particularly, species belonging to genus *Cynoglossus*, *Takifugu* and *Alepes* were less reported in previous studies but were indeed common dominant species along the coast of Fujian [20,31,32]. This underscores the efficacy of eDNA in uncovering hidden biodiversity, complementing traditional survey methods [6,33]. However, our analysis also unveiled spatial variations in fish diversity, with some sites exhibiting significantly lower Shannon–Wiener diversity indices. Similar findings have been reported in other studies, where environmental factors such as tidal fluctuations and water exchange dynamics influence eDNA detection efficacy [34,35]. Notably, there were significant spatial differences in this survey. Consistent with previous hydrodynamic modeling of Sansha Bay [36], we observed the lowest Shannon–Wiener and Simpson diversity indices at the outer-bay sites S10 and S8, which are characterized by the highest water exchange frequencies in the bay. the half-exchange time (*T*_h_) of seawater in this region is less than 10 days, indicating rapid flushing. In contrast, inner-bay sites such as S1 and S8, which are deep within the bay, exhibited significantly higher diversity. These areas are characterized by much slower water exchange, with *T*_h_ exceeding 30–40 days at the bay heads due to weaker residual currents (<0.05 m s^−1^) and complex multi-eddy structures. This allows for greater eDNA accumulation, reflecting a more stable or abundant local fish community. Estuarine sites (S2, S3, and S6) exhibited moderate diversity, likely attributable to osmotic stress on marine taxa from high freshwater runoff, compounded by additional dilution effects from river discharge. These findings align with long-term hydrological monitoring records and underscore the critical role of site-specific hydrodynamics in interpreting eDNA-based biodiversity assessments.

Comparing eDNA with traditional trawl data, it was found that the dominant species in this water area could be well reflected. The top six species ranked by OTUs were indeed the dominant species in Sansha Bay trawl results. At the same time, this study also detected sequences of rare species, such as *A. Japonica*, *Megalaspis cordyla*, *Rachycentron canadum*, and *Omobranchus elegans*. While these species are documented in Fujian Fish Fauna or in other previous studies, their absence in post 21st-century surveys suggested eDNA could capture signals of historically present or low-abundance tax [37]. However, the interpretation of these detections warrants caution. For instance, the detection of *R. glesne* and *A. japonica* does not necessarily confirm their current residency within Sansha Bay. Alternative explanations must be considered, including the transport of extracellular DNA via oceanic currents, the persistence of DNA from decaying remains, potential contributions from aquaculture activities, and the possibility of taxonomic assignment uncertainties or sequencing artifacts [38,39]. Therefore, eDNA detections should be regarded as indicators of potential presence within the recent past, requiring corroboration from morphological surveys or species-specific assays for definitive confirmation [19,40,41]. This aligns with previous research highlighting the limitations of traditional survey methods in detecting cryptic or less abundant species [42].

A notable finding from our study is the disparity between water and sediment eDNA results, indicating distinct fish communities in these habitats. This corroborates previous research highlighting the complementary nature of water and sediment eDNA in capturing different components of fish biodiversity [42,43]. This study also confirmed that rare or overlooked benthic species, particularly those within the order Pleuronectiformes and Tetraodontiformes, were exclusively detected in sediment samples, thereby significantly supplementing the fish diversity catalog. These findings underscore the necessity of integrating eDNA from multiple matrices, including different levels of water column and sediment, to capture species inhabiting different niches across the water column and benthos. Such a comprehensive approach is vital for enhancing the accuracy and reliability of fish diversity assessments in complex coastal ecosystems [44,45]. Several constraints of our sampling design warrant explicit acknowledgment. First, the asymmetric distribution of sediment samples relative to water samples limits our ability to draw spatially comprehensive conclusions about benthic eDNA patterns. This limitation stems from substrate heterogeneity and vessel safety constraints rather than experimental design choice, and future studies should prioritize symmetric sampling across accessible soft-sediment habitats. Second, while 2 L filtration proved sufficient to detect 41 fish species in this high-biomass system, we recognize that larger volumes may be necessary to capture rare taxa in less productive seasons or in more oligotrophic coastal waters. Third, the lack of synchronous environmental metadata precluded quantitative multivariate analyses of environmental drivers. Addressing these limitations in future monitoring campaigns will enhance the accuracy and spatial resolution of eDNA-based biodiversity assessments in complex mariculture bays.

The impact of aquaculture on eDNA detection cannot be ignored, where the annual output of mariculture was 0.66 million tons in 2022 with intensive cages and floating rafts [11,22,46]. The extremely high read abundance of cultured species like *L. crocea* likely creates a substantial eDNA background, potentially masking the signals of less abundant wild species. Feed ingredients, especially the addition of fish meal, can also interfere with rDNA results in aquaculture areas. This underscores the challenge of distinguishing between wild and farmed individuals in intensive mariculture areas, where DNA from cages may dominate the environmental signal. Similar observations have been made in other aquaculture-intensive regions, highlighting the need to account for aquaculture species in eDNA surveys [47,48].

Moving forward, a multi-faceted approach to fish diversity monitoring was recommended, incorporating advanced sampling techniques, integrating water and sediment eDNA analyses, and validating results through traditional surveys. Additionally, local data is indispensable for fish metabarcoding analysis. In conclusion, this study demonstrates that eDNA can effectively analyze the fish diversity composition in this region, while also providing strong supplementary samples that are not easily collected by traditional fishing [49,50,51]. In conclusion, our study demonstrates the efficacy of eDNA in assessing fish diversity in Sansha Bay and provides valuable insights for future conservation and management efforts in the region.

## 5. Conclusions

(1) Integrated surface water and sediment eDNA metabarcoding identified 40 fish species across 16 orders, confirming the value of dual-matrix sampling for comprehensive diversity assessments. (2) Shannon–Wiener diversity indices were generally low across all sites but exhibited clear spatial variation, primarily driven by water exchange rates where outer-bay sites with frequent flushing showed significantly lower diversity than inner bay and estuarine zones. (3) Sediment eDNA sampling is indispensable for future fish diversity surveys, as it exclusively captured all five detected flatfish species and markedly improved detection accuracy of benthic taxa. (4) eDNA and traditional trawl surveys showed strong complementarity, with eDNA detecting 30 species missed by historical trawls. Combined application of both methods is recommended to generate more complete biodiversity inventories. For local fisheries management, we recommend integrating dual-matrix eDNA monitoring into Sansha Bay’s annual fishery resource surveys to better track wild fish population trends, particularly for benthic species and rare taxa masked by intensive *L. crocea* farming.

## Data Availability

The raw eDNA sequencing data generated in this study contain sensitive information. The data are available from the corresponding author (zhizhang@mju.edu.cn) upon reasonable request, subject to these legal restrictions.

## Supplementary Materials

The following supporting information can be downloaded at https://www.mdpi.com/article/10.3390/biology15161336/s1, Table S1: Comparison of species composition in Sansha Bay among four surveys.

## References

[1] Fontoura L., D’agata S., Gamoyo M., Barneche D.R., Luiz O.J., Madin E.M., Eggertsen L., Maina J.M. (2022). Protecting connectivity promotes successful biodiversity and fisheries conservation. Science.

[2] Boussarie G., Bakker J., Wangensteen O.S., Mariani S., Bonnin L., Juhel J.B., Kiszka J.J., Kulbicki M., Manel S., Robbins W.D. (2018). Environmental DNA illuminates the dark diversity of sharks. Sci. Adv..

[3] Dudgeon D., Arthington A.H., Gessner M.O., Kawabata Z., Knowler D.J., Lévêque C., Naiman R.J., Prieur-Richard A.H., Soto D., Stiassny M.L. (2020). Freshwater biodiversity: Importance, threats, status and conservation challenges. Biol. Rev..

[4] Hänfling B., Lawson Handley L., Read D.S., Hahn C., Li J., Nichols P., Blackman R.C., Oliver A., Winfield I.J. (2016). Environmental DNA metabarcoding of lake fish communities reflects long-term data from established survey methods. Mol. Ecol..

[5] Gao J., Zhang J., Pan C., Xu S., Wu Y., Lv W., Hong M., Hu Y., Wang Y. (2025). Assessing Fish Diversity in the Chishui River Using Environmental DNA (eDNA) Metabarcoding. Fishes.

[6] Deiner K., Bik H.M., Mächler E., Seymour M., Lacoursière-Roussel A., Altermatt F., Creer S., Bista I., Lodge D.M., de Vere N. (2017). Environmental DNA metabarcoding: Transforming how we survey animal and plant communities. Mol. Ecol..

[7] Taberlet P., Bonin A., Zinger L., Coissac E. (2018). Environmental DNA: For Biodiversity Research and Monitoring.

[8] Thomsen P.F., Willerslev E. (2015). Environmental DNA—An emerging tool in conservation for monitoring past and present biodiversity. Biol. Conserv..

[9] Djurhuus A., Closek C.J., Kelly R.P., Pitz K.J., Michisaki R.P., Starks H.A., Walz K.R., Andruszkiewicz E.A., Olesin E., Hubbard K. (2020). Environmental DNA reveals seasonal shifts and potential interactions in a marine community. Nat. Commun..

[10] Zhang Z., Wang F., Lei L., Zheng N., Shen Z., Mu J. (2024). Spatio-temporal dynamics of carbonate system during macroalgae farming season in a semi-closed bay from southeast China. Front. Mar. Sci..

[11] Zhang Z., Lei L., Wang F., Li R., Li C., Huang Y., Mu J. (2024). Assessment and spatial pattern of carbon sequestration potential of mariculture in Sansha Bay. Ocean Dev. Manag..

[12] Fang Y., Li M., Liu X., Song Y., Lin M., Yao H. (2024). Response of heavy metal distribution of surface sediments to aquaculture in Sansha Bay, China. J. East China Norm. Univ. (Nat. Sci.).

[13] Yuan J., Lin H., Wu L., Zhuang X., Ma J., Kang B., Ding S. (2021). Resource status and effect of long-term stock enhancement of large yellow croaker in China. Front. Mar. Sci..

[14] Yan L.T., Jiang Y., Xu Q., Ding G.M., Chen X.Y., Liu M. (2022). Reproductive dynamics of the large yellow croaker *Larimichthys crocea* (Sciaenidae), a commercially important fishery species in China. Front. Mar. Sci..

[15] Zheng S., Zhou S., Yang W., Lukwambe B., Nicholaus R., Zhu J., Zheng Z. (2023). Spatial-temporal dynamics of bacterioplankton communities in the breeding area of large yellow croaker *Larimichthys crocea* in Sansha Bay, China. J. Oceanol. Limnol..

[16] Ma W., Dong S., Shi S., Zhang Y., Zhou X., Huang L. (2026). Balancing cage aquaculture and macroalgal cultivation: Scale-dependent management references for eutrophication mitigation in Sansha Bay, China. Aquac. Rep..

[17] Wang Y., Guo X., Wang G., Wang L., Huang T., Li Y., Wang Z., Dai M. (2024). Assessment of nutrient cycling in an intensive mariculture system. Mar. Pollut. Bull..

[18] Dai Y. (2006). Distribution of fish eggs, larva and juveniles in Sansha Bay, Fujian. J. Oceanogr. Taiwan Strait.

[19] Wang Y., Chen K., Gao J., Wang M., Dong J., Xie Y., Giesy J.P., Jin X., Wang B. (2021). Environmental DNA of preservative ethanol performed better than water samples in detecting macroinvertebrate diversity using metabarcoding. Divers. Distrib..

[20] Jiang Y., Lin B.A., He H.Y., Ding G.M., Yan L.T., Zhang G., Liu M., Zheng L.M. (2021). Species composition and assemblages of ichthyoplankton in Sansha Bay, Fujian Province, China. Front. Mar. Sci..

[21] Xie B., Huang J., Huang C., Wang Y., Shi S., Huang L. (2020). Stable isotopic signatures (δ^13^C and δ^15^N) of suspended particulate organic matter as indicators for fish cage culture pollution in Sansha Bay, China. Aquaculture.

[22] Han A., Kao S.J., Lin W., Lin Q., Han L., Zou W., Tan E., Yao L., Ding G., Lin H. (2021). Nutrient budget and biogeochemical dynamics in Sansha Bay, China: A coastal bay affected by intensive mariculture. J. Geophys. Res. Biogeosci..

[23] Song Y., Li M., Fang Y., Liu X., Yao H., Fan C., Tan Z., Liu Y., Chen J. (2023). Effect of cage culture on sedimentary heavy metal and water nutrient pollution: Case study in Sansha Bay, China. Sci. Total Environ..

[24] Miya M., Sato Y., Fukunaga T., Sado T., Poulsen J.Y., Sato K., Minamoto T., Yamamoto S., Araki H., Kondoh M. (2015). MiFish, a set of universal PCR primers for metabarcoding environmental DNA from fishes: Detection of more than 230 subtropical marine species. R. Soc. Open Sci..

[25] Sato Y., Miya M., Fukunaga T., Sado T., Iwasaki W. (2018). MitoFish and MiFish pipeline: A mitochondrial genome database of fish with an analysis pipeline for environmental DNA metabarcoding. Mol. Biol. Evol..

[26] Zhu T., Sato Y., Sado T., Miya M., Iwasaki W. (2023). MitoFish, MitoAnnotator, and MiFish pipeline: Updates in 10 years. Mol. Biol. Evol..

[27] Zhu Y. (1984). Fishes of Fujian.

[28] Oksanen J., Blanchet F.G., Kindt R., Legendre P., Minchin P.R., O’Hara R.B., Solymos P., Stevens M.H.H., Szoecs E., Wagner H. (2024). Vegan: Community Ecology Package. R Package Version 2.6-6.1. https://cran.r-project.org/web/packages/vegan/index.html.

[29] Wang J., Lin Y., Zhou M., Liu Y. (2010). The ecological distribution of fish eggs and larvae in Sansha Bay, Fujian. J. Xiamen Univ. (Nat. Sci.).

[30] Shen C. (2011). Fish community composition and diversity in Sansha Bay of Fujian. Mar. Fish..

[31] Liu C., Xu Q., Wang L., Xing Y., Song J., Lin B., Kang B., Liu M. (2023). Nekton diversity, density, and community structure of spring and autumn in coastal waters of eastern Fujian Province. Biodivers. Sci..

[32] Wang J.Q., Li J., Shih Y.J., Huang L.M., Wang X.R., Chu T.J. (2023). Sustainability perspective of Minjiang estuary coastal fisheries management-Estimation of fish richness. Water.

[33] Bessell T.J., Appleyard S.A., Stuart-Smith R.D., Johnson O.J., Ling S.D., Heather F.J., Lynch T.P., Barrett S.S., Stuart-Smith J. (2023). Using eDNA and SCUBA surveys for detection and monitoring of a threatened marine cryptic fish. Aquat. Conserv..

[34] Jeunen G.J., Knapp M., Spencer H.G., Lamare M.D., Taylor H.R., Stat M., Bunce M., Gemmell N.J. (2019). Environmental DNA (eDNA) metabarcoding reveals strong discrimination among diverse marine habitats connected by water movement. Mol. Ecol. Resour..

[35] Harris M., Brodeur N., LeBlanc F., Douglas S., Chamberland P., Guyondet T., Steeves R., Gagné N. (2022). eDNA and acoustic tag monitoring reveal congruent overwintering distributions of Striped Bass in a hydrologically complex estuarine environment. Fishes.

[36] Lin H., Chen Z., Hu J., Cucco A., Zhu J., Sun Z., Huang L. (2017). Numerical simulation of the hydrodynamics and water exchange in Sansha Bay. Ocean Eng..

[37] Waples R.S., Punt A.E., Cope J.M. (2008). Integrating genetic data into management of marine resources: How can we do it better?. Fish Fish..

[38] Ficetola G.F., Pansu J., Bonin A., Coissac E., Giguet-Covex C., De Barba M., Gielly L., Lopes C.M., Boyer F., Pompanon F. (2015). Replication levels, false presences and the estimation of the presence/absence from eDNA metabarcoding data. Mol. Ecol. Resour..

[39] Carvalho C.S., De Oliveira M.E., Rodriguez-Castro K.G., Saranholi B.H., Galetti P.M. (2022). Efficiency of eDNA and iDNA in assessing vertebrate diversity and its abundance. Mol. Ecol. Resour..

[40] Yamamoto S., Masuda R., Sato Y., Sado T., Araki H., Kondoh M., Minamoto T., Miya M. (2017). Environmental DNA metabarcoding reveals local fish communities in a species-rich coastal sea. Sci. Rep..

[41] Pascher K., Švara V., Jungmeier M. (2022). Environmental DNA-based methods in biodiversity monitoring of protected areas: Application range, limitations, and needs. Diversity.

[42] Valentini A., Taberlet P., Miaud C., Civade R., Herder J., Thomsen P.F., Bellemain E., Besnard A., Coissac E., Boyer F. (2016). Next-generation monitoring of aquatic biodiversity using environmental DNA metabarcoding. Mol. Ecol..

[43] Yamamoto S., Minami K., Fukaya K., Takahashi K., Sawada H., Murakami H., Tsuji S., Hashizume H., Kubonaga S., Horiuchi T. (2016). Environmental DNA as a ‘snapshot’ of fish distribution: A case study of Japanese jack mackerel in Maizuru Bay, Sea of Japan. PLoS ONE.

[44] Holman L.E., de Bruyn M., Creer S., Carvalho G., Robidart J., Rius M. (2019). Detection of introduced and resident marine species using environmental DNA metabarcoding of sediment and water. Sci. Rep..

[45] Pawlowski J., Bruce K., Panksep K., Aguirre F.I., Amalfitano S., Apothéloz-Perret-Gentil L., Baussant T., Bouchez A., Carugati L., Cermakova K. (2022). Environmental DNA metabarcoding for benthic monitoring: A review of sediment sampling and DNA extraction methods. Sci. Total Environ..

[46] Lin H., Chen Z., Hu J., Cucco A., Sun Z., Chen X., Huang L. (2019). Impact of cage aquaculture on water exchange in Sansha Bay. Cont. Shelf Res..

[47] Kelly R.P., O’Donnell J.L., Lowell N.C., Shelton A.O., Samhouri J.F., Hennessey S.M., Feist B.E., Williams G.D. (2016). Genetic signatures of ecological diversity along an urbanization gradient. PeerJ.

[48] Snyder M.R., Stepien C.A., Marshall N.T., Scheppler H.B., Black C.L., Czajkowski K.P. (2020). Detecting aquatic invasive species in bait and pond stores with targeted environmental (e)DNA high-throughput sequencing metabarcode assays: Angler, retailer, and manager implications. Biol. Conserv..

[49] Mauro M., Longo F., Vizzini A., Lo Valvo M., Radovic S., Orecchio G., De Luca R., Luparello C., Mauro A.M., Cuttitta A. (2025). Environmental DNA as a tool for the preliminary assessment of vertebrate biodiversity: A case study from sicilian freshwater ecosystems. Biology.

[50] Wilcox T.M., McKelvey K.S., Young M.K., Sepulveda A.J., Shepard B.B., Jane S.F., Whiteley A.R., Lowe W.H., Schwartz M.K. (2016). Understanding environmental DNA detection probabilities: A case study using a stream-dwelling char *Salvelinus fontinalis*. Biol. Conserv..

[51] Stoeckle M.Y., Adolf J., Charlop-Powers Z., Dunton K.J., Hinks G., VanMorter S.M. (2021). Trawl and eDNA assessment of marine fish diversity, seasonality, and relative abundance in coastal New Jersey, USA. ICES J. Mar. Sci..

