# Supplementary material for: Comprehensive Assessment of Fish Diversity Based on Surface Water and Sediment eDNA—A Case from Sansha Bay, Southeastern China
Source: Biology (Basel). 2026 Aug 7;15(16):1336. doi: 10.3390/biology15161336 (PMC13509325; doi:10.3390/biology15161336)
Supplement: Supplementary file 1 [file biology-15-01336-s001.zip › biology-4440170-supplementary.pdf]

Table S1. Comparison of species composition in Sansha Bay among four surveys

Species	1990	2007-2008	2019	This study
Anguilla japonica				+
Clupanodon punctatus	+	+	+	
Clupanodon thrissa			+	+
Etrumeus micropus	+	+		
Coilia mystus				+
Coilia grayii				+
Sardinella lemuru	+	+		
Setipinna tenuifilis			+	
Engrauli sp.		+		+
Engrauli japonicus	+		+	
Thryssa kammalensis			+	
Thryssa sp.		+		
Scomberomorus niphonius			+	
Ilisha elongata	+			
Stolephorus commersonii	+	+	+	
Allanetta bleekeri	+			
Hyporhamphus intermedius		+	+	
Chanos chanos		+		
Mugil cephalus		+		
Mugilidae sp.	+	+		
Liza haematocheila （*Planiliza haematocheilus*）		+	+	
Planiliza affinis			+	
Liza carinalus		+		
Syngnathus acus		+		
Syngnathus djanong	+			
Syngnathus cyanospilus	+			
Scomber japonicus				+
myctophum pterotum				+
Terapon theraps				+
Pelates quadrilineatus			+	
Hippichthys sp.			+	
Lepidotrigla sp.		+		
Lateolabrax japonicus		+	+	+
Siganus fuscescens				+
Siganus spinus				+
Siganus canaliculatus				+
Sillago japonica	+	+		
Alepes djedaba				+
Alepes kleinii				+

Megalaspis cordyla				+
Caranx malabarious	+			
Trachurus japonicus	+			
Johnius grypotus		+		
Pseudosciaena crocea	+	+	+	+
Larimichthys crocea				+
Nibea albiflora			+	
Atrobucca nibe				+
Collichthys lucidus			+	+
Sciaenidae sp.		+		
Sciaenops ocellatus			+	
Nuchequula nuchalis			+	
Photopectoralis bindus			+	+
Evynnis tumifrons				+
Leiognathus sp.	+	+		
Sparus latus		+		
Sparus macrocephalus		+		
Acanthopagrus schlegelii			+	
Pagrosomus major	+		+	
Rhabdosargus sarba			+	
Sparus aurata			+	
Acropoma japonicum				+
Sparidae sp.		+		
Hapalogenys analis			+	
Hapalogenys nigripinnis			+	
Ambassis gymnocephalus			+	
Epinephelus akaara			+	
Pomadasyidae sp.		+		
Halichoeres tenuispinis			+	
Labridae sp.		+		
Callionymidae sp.		+		
Blenniidae sp.			+	
Parablennius yatabei（*Blennius yatabei*）		+	+	
Omobranchus sp.		+		
Omobranchus elegans	+			+
Omobranchus punctatus			+	
Rachycentron canadum			+	+
Regalecus glesne				+
Eupleurogrammus muticus		+		
Trichiurus haumela	+			
Acentrogobius caninus			+	

Acentrogobius viganensis			+	+
Istigobius campbelli				+
Amblychaeturichthys hexanema			+	
Rhinogobius similis			+	
Rhinogobius sp.			+	
Amoya chusanensis （*Ctenogobiu*）			+	
Aulopareia unicolor			+	
Mugilogobius abei			+	
Odontamblyopus lacepedii			+	
Oxuderces dentatus			+	
Parachaeturichthys polynema		+	+	+
Tridentiger barbatus			+	
Tridentiger trigonocephalus			+	
Tridentiger bifasciatus			+	
Trypauchen vagina			+	
Glossogobius giuris		+		
Taenioides anguillaris			+	
Acanthogobius hasta				+
Gobiidae sp.	+	+	+	
Boleophthalmus pectinirostris			+	
Periophthalmus modestus			+	
Scartelaos gigas			+	
Bostrychus sinensis				+
Eleotris oxycephala				+
Butis koilomatodon			+	
Sebastiscus marmoratus		+	+	
Scorpaenidae sp.		+		
Unimicus japonicus	+			
Platycephalus indicus		+	+	
Solea ovata			+	
Cynoglossus robustus	+	+		
Cynoglossus gracilis				+
Cynoglossus joyneri			+	
Cynoglossus abbreviatus				+
Cynoglossus sp.	+	+		
Paraplagusia japonica				+
Soleidae sp.		+		
Bothidae sp.	+			
Takifugu sp.		+		
Takifugu fasciatus				+
Takifugu alboplumbeus				+
Takifugu bimaculatus				+

Takifugu xanthopterus			+	+
Fugu oblongus	+	+		
***Gastrophysus* sp.**	+	+		
Lagocephalus gloveri				+
Plectropomus leopardus				+
Scatophagus argus				+
**references**	Dai, 2006 [18]	Wang et al.,2010 [29]; Shen, 2011 [30]	Jiang et al., 2021[20]	This study
